# Novel 4-Thiazolidinone Derivatives as Anti-Infective Agents: Synthesis, Characterization, and Antimicrobial Evaluation

**DOI:** 10.1155/2016/8086762

**Published:** 2016-01-26

**Authors:** Amit Gupta, Rajendra Singh, Pankaj K. Sonar, Shailendra K. Saraf

**Affiliations:** Faculty of Pharmacy, Babu Banarasi Das Northern India Institute of Technology, Sector-II, Dr. Akhilesh Das Nagar, Faizabad Road, Lucknow, Uttar Pradesh 227105, India

## Abstract

A series of new 4-thiazolidinone derivatives was synthesized, characterized by spectral techniques, and screened for antimicrobial activity. All the compounds were evaluated against five Gram-positive bacteria, two Gram-negative bacteria, and two fungi, at concentrations of 50, 100, 200, 400, 800, and 1600 *µ*g/mL, respectively. Minimum inhibitory concentrations of all the compounds were also determined and were found to be in the range of 100–400 *µ*g/mL. All the compounds showed moderate-to-good antimicrobial activity. Compounds** 4a** [2-(4-fluoro-phenyl)-3-(4-methyl-5,6,7,8-tetrahydro-quinazolin-2-yl)-thiazolidin-4-one] and** 4e** [3-(4,6-dimethyl-pyrimidin-2-yl)-2-(2-methoxy-phenyl)-thiazolidin-4-one] were the most potent compounds of the series, exhibiting marked antimicrobial activity against* Pseudomonas fluorescens*,* Staphylococcus aureus,* and the fungal strains. Thus, on the basis of results obtained, it may be concluded that synthesized compounds exhibit a broad spectrum of antimicrobial activity.

## 1. Introduction

Infections caused by microbes are among the leading causes of death worldwide. The availability of limited number of antibiotics for the treatment of infections, and continuous development of resistance to the recently used antimicrobial agents, pose a serious challenge [1]. Thus, the discovery of innovative and potent antimicrobial agents may be the only way to resolve the resistance problem and develop successful remedy for the treatment of infectious diseases. 4-Thiazolidinones have recently been reported to be novel inhibitors of the bacterial enzyme Mur B (a precursor during the biosynthesis of peptidoglycan) and also to block some pathogenic mechanisms of bacteria [2]. 4-Thiazolidinones are derivatives of thiazolidine with a carbonyl group at the fourth position. This is a core structure in various synthetic pharmaceuticals displaying a broad spectrum of biological activities such as antimycobacterial [3–5], antimicrobial [6–19], anticancer [20, 21], anticonvulsant [22–32], anti-inflammatory and analgesic [33–37], antiparasitic [38–43], antiviral and anti-HIV [44–49], antidiabetic [50–52], antihypertensive [53–55], antihyperlipidemic [56–58], and MAO inhibitors [59]. The substituted thiazolidine moiety has attracted considerable interest in the development of biologically active compounds. In the present study, novel arylidene substituted 4-thiazolidinones were synthesized and evaluated as antimicrobial agents from heterocyclic scaffold.

## 2. Materials and Methods

All the chemicals and solvents used in the study were procured from S. D. Fine-Chem. Ltd., Mumbai, and Sigma-Aldrich Chemie, Germany. Culture media for antimicrobial screening were procured from HiMedia Laboratories, Mumbai. The standard microbial strains were procured from Microbial Type Culture Collection (MTCC), Institute of Microbial Technology, Chandigarh, India. Spectral studies (IR, NMR, and mass spectrometry, Table 1) of the synthesized compounds were performed at Central Drug Research Institute, Lucknow.

### 2.1. Chemistry

**Figure 1:**
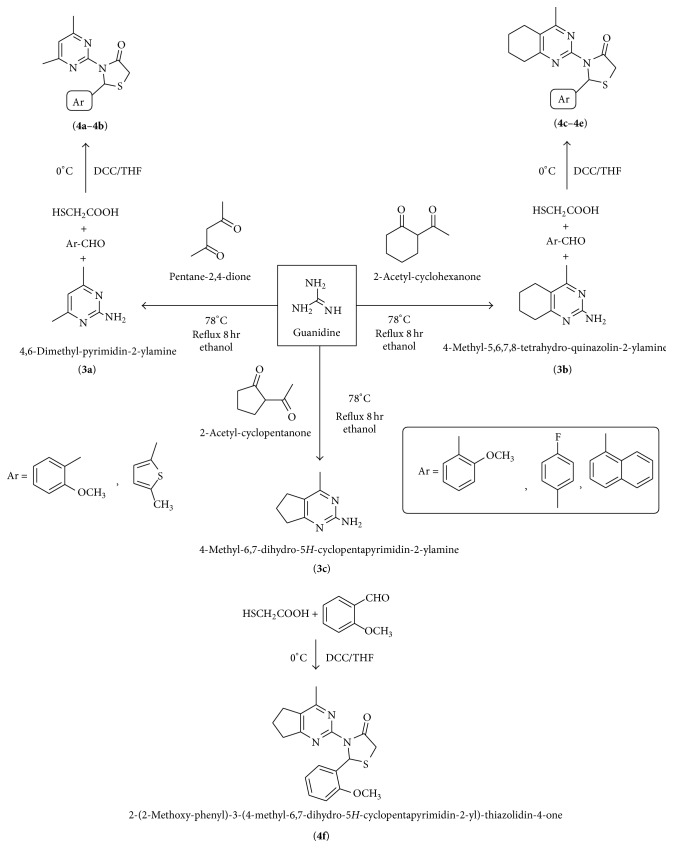
Synthetic pathway for the compounds (**4a**–**4f**).

4-Thiazolidinones were synthesized in two steps. In the first step, 2-aminopyrimidine derivatives were synthesized by the reaction of 1,3-dicarbonyl compounds with guanidine. Final compounds (**4a**–4**f**) were synthesized by the reaction of compounds of step 1 with substituted aromatic aldehyde (s) and mercaptoacetic acid (s), using DCC as intramolecular cyclizing agent (Figure 1).

#### 2.1.1. General Procedure for the Synthesis of Compounds (**3a**–**3c**)

Equimolar solution of dicarbonyl compounds and guanidine in ethanol was refluxed at 78°C for 8 hr. The reaction mixture was then concentrated to dryness under reduced pressure and the residue was partitioned in ethyl acetate. The organic layer was successively washed with water and then finally with brine. The organic layer was dried over sodium sulphate and the solvent was removed under reduced pressure to get the products (**3a**–**3c**) [49]. The progress of the reaction was monitored by TLC, using 5% methanol in chloroform.

#### 2.1.2. General Procedure for the Synthesis of Compounds (**4a**–**4f**)

A solution of** 3a**–**3c** (10 mmol) and various substituted aldehydes (20 mmol) was stirred in THF, under ice cold conditions for 5 min, followed by the addition of mercaptoacetic acid (30 mmol). After 5 min, DCC (12 mmol) was added to the reaction mixture at 0°C and the reaction mixture stirred for an additional 5 hr at room temp. DCU was removed by filtration, the filtrate was concentrated to dryness under reduced pressure, and the residue was extracted with ethyl acetate. The organic layer was successively washed with 5% aqueous citric acid, water, and 5% aqueous sodium hydrogen carbonate and then finally with brine. The organic layer was dried over sodium sulphate and the solvent was removed under reduced pressure to get the products (**4a**–**4f**) [60]. The progress of the reaction was monitored by TLC, using the solvent system methanol : chloroform (2 : 98).

### 2.2. Antimicrobial Screening

#### 2.2.1. Test Microorganisms

Antimicrobial activity of the synthesized compounds was studied against nine microorganisms, including seven bacterial strains—*Bacillus subtilis* (MTCC 441),* Staphylococcus aureus* (MTCC 1430),* Pseudomonas aeruginosa* (MTCC 424),* Bacillus pumilus* (MTCC 1456),* Pseudomonas fluorescens* (MTCC 2421),* Escherichia coli *(MTCC 1573), and* Micrococcus luteus *(MTCC 1538)—and two fungal strains,* Aspergillus niger *(MTCC 2546) and* Penicillium chrysogenum *(MTCC 161).

#### 2.2.2. Preparation of the Samples and Standard Solution

The compounds (**4a–4f**) were dissolved in 10% DMSO at the concentrations of 50, 100, 200, 400, 800, and 1600 *µ*g/mL, respectively. Norfloxacin and fluconazole, used as the standard drugs for antibacterial and antifungal studies, respectively, were also dissolved in 10% DMSO at the concentrations of 10 *µ*g/mL.

#### 2.2.3. Method

Antimicrobial activity of the synthesized compounds was evaluated by cup-plate method. Nutrient broth suspension of test microorganism (10 mL) was added to 100 mL of sterile molten nutrient agar growth media (cooled to 45°C), mixed well, and poured on to sterile petri plates. The agar was allowed to solidify and was then punched to make six wells/cups, using a 6 mm sterile cork borer (separate borer for each organism), to ensure proper distribution of wells in the periphery and one well in the centre. Agar plugs were removed and 50 *µ*L solution of test samples (each compound in six concentrations) was poured into the corresponding marked well using micropipette. Triplicate plates of each organism were prepared. The plates were left at room temperature for 2 h to allow diffusion of samples and then incubated face upward, at corresponding temperature of each organism, for 48 h [61]. The diameters of zone of inhibition were measured to the nearest millimeter (the cup size also included) and are presented in Table 2.

#### 2.2.4. Determination of Minimum Inhibitory Concentration (MIC)

A series of glass tubes, containing different concentrations of the synthesized compounds (in 10% DMSO), with nutrient broth was inoculated with the required quantity of the inoculums to obtain a suspension of microorganisms which contained 10^5^ colony forming units per milliliter. One growth control tube was prepared without the addition of the compounds or the microorganisms. The tubes were incubated at 37°C for 24 h. The turbidity produced in each tube was recorded on a UV-visible spectrometer [62, 63]. The observed MICs (*µ*g/mL) are presented in Table 3.

## 3. Results and Discussion

4-Thiazolidinones were synthesized in two steps. In the first step, 2-aminopyrimidine derivatives were synthesized by the reaction of 1,3-dicarbonyl compounds with guanidine. Finally, the compounds (**4a**–**4f**) were synthesized by reaction of the compounds of step 1 with substituted aromatic aldehydes and mercaptoacetic acids, using DCC as intramolecular cyclizing agent.

Characteristic peaks were observed for N-H stretching, C=O stretching, and C-N stretching. The IR spectra of the 4-thiazolidinone derivatives exhibited C=O lactam amide stretching vibration in the range of 1637–1728 cm^−1^. [M]^+^/[M + 1]^+^ peaks were observed for the synthesized compounds. ^1^H-NMR spectra of the compounds indicated the presence of two diastereotopic protons at C-5 position and one single proton at C-2 position; doublets were obtained in the region of 3.07–3.47 ppm. A doublet integrated for one proton appeared at the *δ* value of 2.37–2.74 ppm. This can be attributed to the C-2 proton of the 4-thiazolidinone ring.

The antimicrobial activity was observed at 50, 100, 200, 400, 800, and 1600 *µ*g/mL, respectively (Table 2). Minimum inhibitory concentrations of the synthesized compounds were also determined, in nutrient broth by tube dilution method. MICs were in the range of 100–500 *µ*g/mL, which were recorded as the optical density, at 530 nm.

The antimicrobial screening revealed that all the synthesized compounds possessed a wide spectrum of antimicrobial profile against the tested microbial strains. The compounds, which were active against bacterial and fungal strains, were effective at a much higher concentration than the standard drugs norfloxacin and fluconazole. All the compounds exhibited good-to-moderate antimicrobial activity against all the strains. Compounds** 4b**,** 4c**, and** 4d** were found to be more effective against the fungal strains than the bacterial strains. On the basis of MIC values of the synthesized compounds, the order of antimicrobial spectrum was** 4b > 4a > 4d > 4c > 4f > 4e**. Compound 2-(4-fluoro-phenyl)-3-(4-methyl-5,6,7,8-tetrahydro-quinazolin-2-yl)-thiazolidin-4-one (**4a**) and compound 3-(4,6-dimethyl-pyrimidin-2-yl)-2-(2-methoxy-phenyl)-thiazolidin-4-one (**4e**) were the most potent compounds of the series, exhibiting marked antibacterial activity against* Pseudomonas fluorescens* and* Staphylococcus aureus*.

## 4. Conclusion

In the present study, six new 4-thiazolidinone derivatives were synthesized, characterized, and evaluated for their antimicrobial potential. The compounds exhibited antimicrobial activity against the selected Gram-positive and Gram-negative bacterial strains and the fungal strains. Overall, 2-(4-fluoro-phenyl)-3-(4-methyl-5,6,7,8-tetrahydro-quinazolin-2-yl)-thiazolidin-4-one and 3-(4,6-dimethyl-pyrimidin-2-yl)-2-(2-methoxy-phenyl)-thiazolidin-4-one were found to be the most potent members of the series. On the basis of the antimicrobial activity studies, it may be concluded that all the compounds have a broad spectrum of antimicrobial activity.

Thus, the study provides a lead for the syntheses and evaluation of more 4-thiazolidinone derivatives for antimicrobial activity, as the same could lead to the discovery of some promising agents.

## Figures and Tables

**Table 1:** Physical and spectral characterization of the synthesized compounds (**4a–4f**).

Comp.	Melting range	% yield (w/w)	IR (KBr) cm^−1^	Mass *m/z* [M + 1]^+1^	^1^H NMR (*δ* ppm)
**4a**	Viscous liquid	32.15	1728.1, 3453.60, 1217.2	345	2.37, 3.47, 7.60–6.46

**4b**	Viscous liquid	59.89	1637.4, 3445.9, 1216.40	374	2.04–2.74, 3.18–3.95, 7.5–6.4

**4c**	128–130°C	40.11	1711.4, 3418.6, 1216.4 763.7	356	2.73, 3.32, 3.93, 4.35 7.85–6.88

**4d**	178–180°C	43.04	1691.9, 3296.7, 1592.6	340	1.89, 2.50, 3.07, 4.28 7.87–6.87

**4e**	114–116°C	59.80	1584.2, 3427.9, 1216.4 1707.4	316	1.25, 3.67, 3.33, 4.29 7.85–6.56

**4f**	Viscous liquid	37.48	1663.20, 3021.20, 1217.00	306	1.91, 2.56, 3.33, 5.09 6.92–6.80

**Table 2:** Mean diameter of zone of inhibition (mm) of synthesized compounds **(4a–4f)**, standard and control against various microorganisms.

S. number	Compounds	Conc. (*μ*g/mL)	Gram +ve strains				Gram –ve strains			Fungal strains	
			SA	BS	BP	ML	PA	PF	EC	AN	PC
1	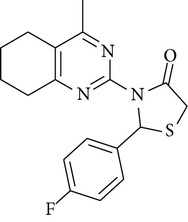 (**4a**-C_18_H_18_FN_3_OS)	50 100 200 400 800 1600	8 9 10 10 11 14	10 11 11 12 13 14	8 9 12 16 18 20	10 10 12 13 14 14	8 10 13 14 16 18	14 14 16 17 18 19	8 10 12 13 13 14	8 10 11 12 13 14	9 10 11 13 15 17

2	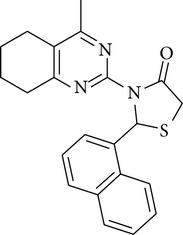 (**4b**-C_22_H_21_N_3_OS)	50 100 200 400 800 1600	8 13 14 15 16 18	12 13 15 16 17 19	13 14 15 17 18 20	15 16 17 18 19 21	12 13 14 15 17 19	12 13 15 16 17 19	8 12 14 15 16 18	12 14 16 17 18 19	12 13 14 15 17 18

3	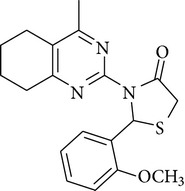 (**4c**-C_19_H_21_N_3_O_2_S)	50 100 200 400 800 1600	8 8 8 8 10 12	8 8 12 14 15 16	8 10 14 16 18 20	8 8 10 13 14 14	9 12 13 14 15 16	10 12 14 15 16 18	10 10 13 14 14 15	8 8 10 12 13 14	10 11 13 15 16 17

4	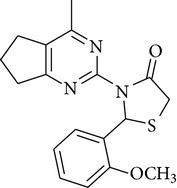 (**4d**-C_18_H_19_N_3_O_2_S)	50 100 200 400 800 1600	8 13 15 17 19 21	13 14 15 17 18 19	12 13 14 16 17 18	14 16 17 18 19 20	15 16 17 18 19 20	13 14 15 16 17 19	12 13 15 16 18 20	8 12 13 14 16 18	8 12 14 16 17 19

5	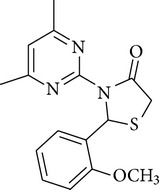 (**4e**-C_16_H_17_N_3_O_2_S)	50 100 200 400 800 1600	12 13 14 15 16 17	14 15 16 20 21 24	12 13 15 16 18 19	13 15 16 17 19 20	8 13 14 15 17 19	8 13 14 16 17 18	8 13 14 15 17 19	8 13 14 15 17 18	12 13 14 16 17 19

6	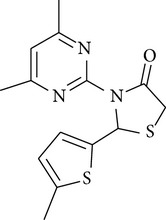 (**4f**-C_14_H_15_N_3_OS_2_)	50 100 200 400 800 1600	8 9 10 12 13 15	9 10 12 14 16 18	8 8 10 12 18 21	8 8 8 10 12 14	10 12 16 18 20 21	10 14 16 17 18 19	8 10 12 14 14 16	8 8 10 12 14 17	8 10 12 13 15 18

7	Norfloxacin	10	25	22	30	24	26	25	27	—	—

8	Fluconazole	10	—	—	—	—	—	—	—	22	23

9	Control (10% DMSO)	—	—	—	—	—	—	—	—	—	—

BS: *B. subtilis*, SA: *S. aureus*, BP: *B. pumilus*, ML: *M. luteus*, PA: *P. aeruginosa*, EC: *E. coli,* PF: *P. fluorescens*, AN: *A. niger*, PC: *P. chrysogenum*, control = 10% v/v DMSO, and (—) = no activity.

**Table 3:** Values of the minimum inhibitory concentration of the synthesized compounds and reference standards.

S. number	Microbial strains	MIC of compounds (*µ*g/mL)							
		**4a**	**4b**	**4c**	**4d**	**4e**	**4f**	N	F
1	*Staphylococcus aureus*	300	500	300	400	400	300	2.5	—
2	*Bacillus subtilis*	300	200	400	300	300	100	5	—
3	*Bacillus pumilus*	300	100	300	100	200	500	1.25	—
4	*Micrococcus luteus*	300	500	500	300	300	300	—	—
5	*Pseudomonas aeruginosa*	200	300	300	300	400	400	2.5	—
6	*Pseudomonas fluorescens*	100	100	300	100	200	300	2.5	—
7	*Escherichia coli*	300	100	300	400	400	200	2.5	—
8	*Aspergillus niger*	300	100	100	100	300	300	—	2.5
9	*Penicillium chrysogenum*	400	100	100	100	300	300	—	1.25

N: norfloxacin and F: fluconazole.
